# Anti-Inflammatory Effects of Endogenously Released Adenosine in Synovial Cells of Osteoarthritis and Rheumatoid Arthritis Patients

**DOI:** 10.3390/ijms22168956

**Published:** 2021-08-19

**Authors:** Rebecca Sohn, Marius Junker, Andrea Meurer, Frank Zaucke, Rainer H. Straub, Zsuzsa Jenei-Lanzl

**Affiliations:** 1 Dr. Rolf M. Schwiete Research Unit for Osteoarthritis, Department of Orthopedics (Friedrichsheim), University Hospital Frankfurt, Goethe University, 60528 Frankfurt/Main, Germany; rebecca.sohn@kgu.de (R.S.); marius.junker@kgu.de (M.J.); andrea.meurer@kgu.de (A.M.); frank.zaucke@kgu.de (F.Z.); 2 Laboratory of Experimental Rheumatology and Neuroendocrine Immunology, Department of Internal Medicine, University Hospital Regensburg, 93053 Regensburg, Germany; rainer.straub@ukr.de

**Keywords:** adenosine, inosine, rheumatoid arthritis, osteoarthritis, synoviocytes, inflammation

## Abstract

Exogenous adenosine and its metabolite inosine exert anti-inflammatory effects in synoviocytes of osteoarthritis (OA) and rheumatoid arthritis (RA) patients. We analyzed whether these cells are able to synthesize adenosine/inosine and which adenosine receptors (ARs) contribute to anti-inflammatory effects. The functionality of synthesizing enzymes and ARs was tested using agonists/antagonists. Both OA and RA cells expressed CD39 (converts ATP to AMP), CD73 (converts AMP to adenosine), ADA (converts adenosine to inosine), ENT1/2 (adenosine transporters), all AR subtypes (A_1_, A_2A_, A_2B_ and A_3_) and synthesized predominantly adenosine. The CD73 inhibitor AMPCP significantly increased IL-6 and decreased IL-10 in both cell types, while TNF only increased in RA cells. The ADA inhibitor DAA significantly reduced IL-6 and induced IL-10 in both OA and RA cells. The A_2A_AR agonist CGS 21680 significantly inhibited IL-6 and induced TNF and IL-10 only in RA, while the A_2B_AR agonist BAY 60-6583 had the same effect in both OA and RA. Taken together, OA and RA synoviocytes express the complete enzymatic machinery to synthesize adenosine/inosine; however, mainly adenosine is responsible for the anti- (IL-6 and IL-10) or pro-inflammatory (TNF) effects mediated by A_2A_- and A_2B_AR. Stimulating CD39/CD73 with simultaneous ADA blockage in addition to TNF inhibition might represent a promising therapeutic strategy.

## 1. Introduction

Rheumatoid arthritis (RA) is a systemic, chronic inflammatory autoimmune disorder affecting around 1% of the population worldwide [1]. RA results in symmetric primary inflammatory polyarthritis characterized by painful swelling in multiple joints [2]. Uncontrolled active synovial inflammation in RA results in joint damage and causes chronic pain, disabilities, decreased quality of life, as well as different comorbidities such as cardiovascular, gastrointestinal, renal and pulmonary diseases or fatigue [3]. To date, RA remains an incurable disease; mainly symptomatic treatment exists [4], although significant progress in knowledge about its pathogenesis has been achieved in recent years.

After RA induction by an initiating factor such as infection or environmental exposure (e.g., smoking or chronic stress) [5], synovial lining cells become activated and start to release matrix-degrading enzymes, pro-inflammatory cytokines and chemokines, which in turn recruit immune cells [6]. A vicious cycle arises, resulting in synoviocyte- and osteoclast-mediated cartilage and bone destruction [7]. Synovial cells in inflamed synovium contain fibroblasts, macrophages, B- and T lymphocytes and dendritic cells, which release different cytokines [8,9]. Key cytokines driving inflammatory processes in RA synovium are IL-6 and TNF. The major IL-6 producers are synovial fibroblasts and pro-inflammatory M1 macrophages. The more anti-inflammatory IL-10 is synthesized mainly by B cells and anti-inflammatory M2 macrophages, while highly pro-inflammatory cytokine TNF is released by T cells, monocytes, and M1 macrophages [10,11].

Recent symptomatic RA therapies are primarily based on disease-modifying antirheumatic drugs (DMARDs), which are often combined with biologicals such as TNF inhibitors that target synovitis and systemic inflammation [12]. However, in many cases, the clinically active disease persists in spite of RA-modifying medications [3,13]. Therefore, alternative therapy options have to be developed. In contrast to RA, osteoarthritis (OA) is mainly a chronic degenerative and not primary inflammatory disorder of the joints even though a low-grade inflammation of the synovium was described [14]. Therefore, OA synoviocytes are often used as a control in studies analyzing inflammatory processes in RA cell cultures.

Previous studies demonstrated that the nucleoside adenosine and its metabolite inosine exert anti-inflammatory effects in different tissues in the human body, such as the lung, liver or kidney after binding to specific G protein-coupled adenosine receptors (Ars) [15,16]. In general, four AR subtypes do exist (A_1_, A_2A_, A_2B_ and A_3_) [17,18,19]. All four subtypes have been detected in the synovial tissue of RA patients in earlier studies [20], and indeed, exogenously applied adenosine and inosine exhibited anti-inflammatory effects in synovial cells from rheumatoid arthritis (RA) patients in vitro [21]. Similar anti-inflammatory effects were reported in murine models of experimental RA by mainly reducing TNF, but also inhibiting IL-1β, and IL-8 release. The majority of these effects were shown to be mediated by the AR subtypes A_2A_, A_2B_ or A_3_ [22,23,24,25].

However, most cell culture studies were performed under 20% O_2_ concentration, which represents neither a physiological nor a pathophysiological microenvironment. In a healthy synovial tissue, O_2_ concentrations of 2–4% predominate, while an inflamed synovium contains only 1–3% O_2_ [26]. The anti-inflammatory effect of applied adenosine or inosine, as well as of specific receptor subtype agonists targeting A_2A_AR and A_3_AR during RA manifestation, was also confirmed in a few animal studies [23,27]. Thus, in these earlier studies, the anti-inflammatory acting adenosine, inosine, or AR agonists/antagonists were applied as a drug externally. However, the human body itself is capable of producing adenosine and inosine [28], and one might use the internal source for experiments.

Different cell types such as cardiomyocytes, circulating lymphocytes, or cancer-associated fibroblasts in the lung are able to convert ATP to AMP by CD39 followed by AMP conversion to adenosine by CD73 both intra- and extracellularly [29]. Regarding RA, only a few studies exist that describe the fact that regulatory T cells in the synovial fluid express CD73 [30], or M2 macrophages in the blood co-express CD39/CD73 [31]. OA synovial tissue and fluid have not yet been investigated in this regard. Moreover, the transmembrane Equilibrative Nucleoside Transporters ENT1 and ENT2 are responsible for adenosine transport between intra- and extracellular space, thereby influencing the extracellularly acting levels of adenosine [32]. Additionally, last but not least, the enzyme adenosine deaminase (ADA), which catalyzes the conversion of adenosine to inosine, belongs to the machinery regulating nucleoside homeostasis [33].

ADA was detected in the synovial fluid of OA and RA patients as well as in RA synovial fibroblasts [34]. However, the existence of these enzymes and transporters has not yet been investigated in mixed synovial cell cultures containing fibroblasts, macrophages, lymphocytes, and dendritic cells [8].

Thus, at present, no study is available demonstrating that OA and RA mixed synoviocytes do express all enzymes of adenosine and inosine synthesis. It is also unclear how, and via which ARs, endogenously released nucleosides could contribute to possible anti-inflammatory events in the synovium. Therefore, we analyzed the entire adenosine synthesizing and transporting machinery in OA and RA mixed synovial cell culture.

## 2. Results

### 2.1. CD39/CD73 and ADA Expression as well as Adenosine/Inosine Release

**Figure 1:**
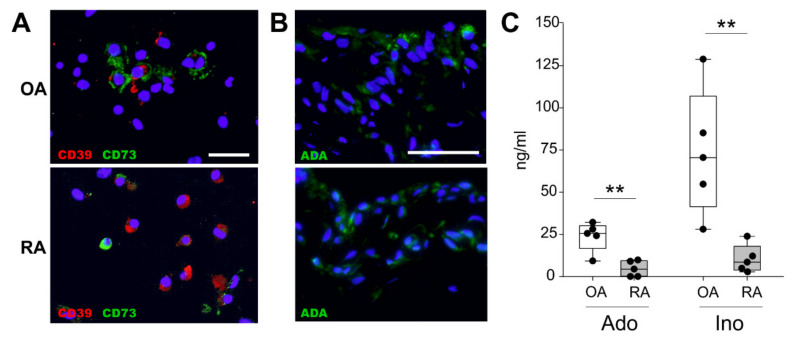
CD39/CD73 expression, ADA expression, and adenosine/inosine release. (**A**) Representative fluorescent micrographs of OA and RA mixed synoviocytes immunostained for CD39 (red) and CD73 (green) and counterstained nuclei with DAPI (blue) (bar: 25 µm). (**B**) Representative fluorescent micrographs of OA and RA mixed synoviocytes immunostained for ADA (green) and counterstained nuclei with DAPI (blue) (bar: 50 µm). (**C**) Quantification of spontaneous adenosine and inosine release in OA and RA mixed synoviocytes. Data are represented as box plots, where the boxes represent the 25th to 75th percentiles, the lines within the boxes represent the median, and the lines outside the boxes represent the 10th and 90th percentiles. Each black circle represents the synovial cells of an individual patient (n = 5). Significant *p*-values are presented as ** *p* ≤ 0.01. Abbreviations: ADA—adenosine deaminase; Ado—adenosine; DAPI—4′,6-diamidino-2-phenylindole; Ino—inosine; OA—osteoarthritis; RA—rheumatoid arthritis.

In order to analyze whether synoviocytes are capable of synthesizing adenosine and inosine, cells were examined for the presence of CD39 and CD73. Double immunostaining revealed that mixed synoviocytes from both OA and RA patients co-expressed CD39 and CD73 (Figure 1A). In order to investigate whether the cells are able to convert adenosine to its metabolite inosine, ADA was stained immunohistochemically. Both OA and RA synovial cells expressed ADA without any differences between the groups (Figure 1B). In addition, after cultivation for 24 h under hypoxia, synovial cells spontaneously released physiologically relevant amounts of adenosine and inosine; however, OA synoviocytes synthesized significantly higher nucleoside concentrations compared to RA cells (mean ± SEM: adenosine: OA 23.8 ± 8.7 ng/mL, RA 4.6 ± 2.7 ng/mL, *p* = 0.008; inosine: OA 73.4 ± 37.5 ng/mL, RA 10.4 ± 8.3 ng/mL, *p* = 0.002) (Figure 1C). No age- or gender-dependent differences were detected.

### 2.2. ENT1/2 Expression

**Figure 2:**
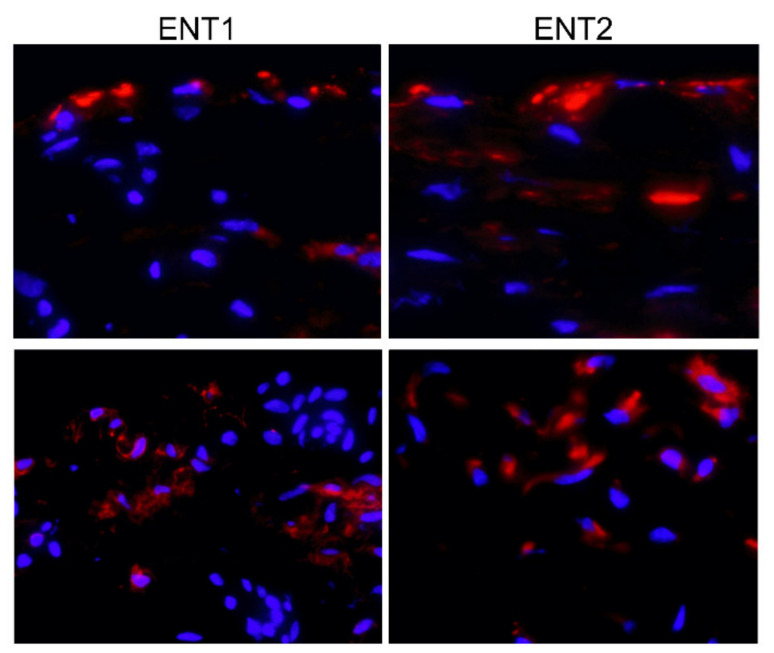
ENT1/2 expression in the synovium. Representative fluorescent micrographs of OA and RA synovial tissues immunostained for ENT1 and ENT2 (red) and counterstained for nuclei with DAPI (blue) (bar: 50 µm). Abbreviations: DAPI—4′,6-diamidino-2-phenylindole; ENT—equilibrative nucleoside transporter; OA—osteoarthritis; RA—rheumatoid arthritis.

To investigate the ability of synovial tissue to transport endogenous adenosine across the cell membrane, immunohistochemical staining of the respective transporters was performed. Both OA and RA synovial cells possess these transporters (ENT1 and ENT2) without any obvious differences between the groups (Figure 2).

### 2.3. Expression of AR Subtypes

**Figure 3:**
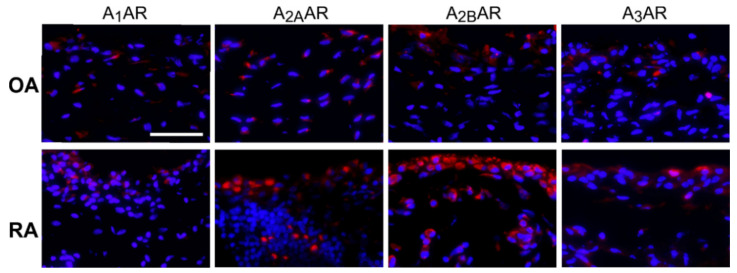
Expression of adenosine receptors (ARs) in the synovium. Representative fluorescent micrographs of OA and RA synovial tissues immunostained for A_1_-, A_2A_-, A_2B_-, and A_3_AR (red) and counterstained for nuclei with DAPI (blue) (bar: 50 µm). Abbreviations: see legends to Figure 1.

In order to determine the possible ability to respond to adenosine, synovial tissue was screened immunohistochemically for all different AR subtypes. In general, all AR subtypes were detectable in OA and RA synovium (Figure 3). A_1_- and A_3A_R expression levels were similar in OA and RA, while the expression of A_2A_- and A_2B_AR seemed to be more pronounced in RA tissue (Figure 3). No further AR quantification was performed, because earlier studies reported and quantified the same phenomenon, namely a dominant A_2B_AR expression in the synovium of RA patients [20].

### 2.4. Effects of CD73 and ADA Inhibition

To better understand the role of endogenously synthesized adenosine within the synovium, its last synthesis step, as well as its conversion to inosine, were blocked by treating mixed synovial cells with the CD37 inhibitor AMPCP or the ADA inhibitor DAA, respectively. The absolute levels of released cytokine concentrations in untreated control groups were as follows: OA IL-6 205.5 ± 72.9 μg/mL, TNF 348.7 ± 135.3 pg/mL, IL-10 254.0 ± 179.2 pg/mL; RA IL-6 364.5 ± 174.4 μg/mL, TNF 580.2 ± 387.9 pg/mL, IL-10 455.7 ± 235.2 pg/mL. Although RA cells seem to release higher cytokine concentrations, the differences between OA and RA cells were not significant. No age- or gender-dependent differences were detected.

**Figure 4:**
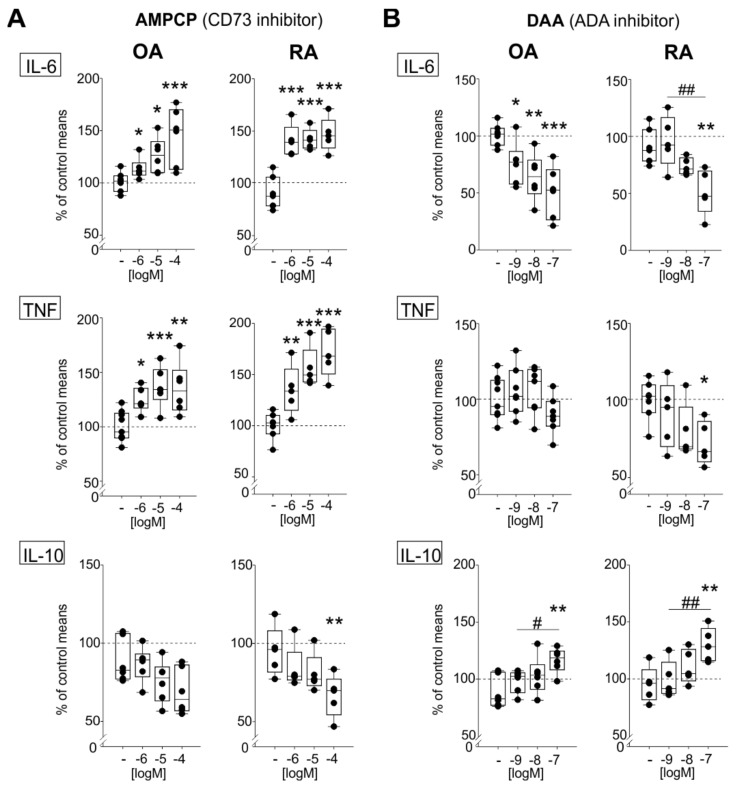
Effects of CD73 and ADA inhibition. (**A**) IL-6, TNF, and IL-10 release in OA and RA mixed synoviocyte cultures after CD37 inhibition using AMPCP (10^−6^ M to 10^−4^ M). (**B)** IL-6, TNF, and IL-10 release in OA and RA mixed synoviocyte cultures after ADA inhibition using DAA (10^−9^ M to 10^−7^ M). Data are represented as box plots (as explained in the legend of Figure 1). Values are expressed as percent of untreated control (untreated control is represented as “-“; the mean of untreated controls is represented as dashed line = 100%). Significant *p*-values are presented as * *p* ≤ 0.05 ** *p* ≤ 0.01 *** *p* ≤ 0.001 when compared to untreated controls or as # *p* ≤ 0.05 ## *p* ≤ 0.01 when two treatment groups were compared. Abbreviations: see legends to Figure 1 and Figure 2.

The treatment of both OA and RA cells with the CD37-blocker AMPCP resulted in significantly increased IL-6 concentrations compared to untreated control cells (OA: 10^−6^ M *p* = 0.036, 10^−5^ M *p* = 0.013, 10^−4^ M *p* < 0.001; RA: 10^−6^ M *p* < 0.001, 10^−5^ M *p* < 0.001, 10^−4^ M *p* < 0.001) (Figure 4A). The same treatment conditions also significantly increased TNF release in OA and RA cells compared to the control (OA: 10^−6^ M *p* = 0.024, 10^−5^ M *p* = 0.001, 10^−4^ M *p* = 0.002; RA: 10^−6^ M *p* = 0.008, 10^−5^ M *p* < 0.001, 10^−4^ M *p* < 0.001) (Figure 4A). In contrast, IL-10 concentrations decreased after applying 10^−4^ M AMPCP in RA (*p* = 0.043), but not in OA mixed synoviocytes (Figure 4A).

Treatment with the adenosine deaminase blocker DAA significantly reduced IL-6 release in OA synovial cells, while in RA cells, only the highest DAA concentration, 10^−7^ M, caused a significant IL-6 reduction (OA: 10^−9^ M *p* = 0.022, 10^−8^ M *p* = 0.004, 10^−7^ M *p* < 0.001; RA: 10^−7^ M *p* = 0.003, 10^−9^ M compared to 10^−7^ M *p* = 0.003) (Figure 4B). Compared to untreated controls, TNF levels in OA and RA mixed synoviocytes were only slightly affected; the only significant reduction was observed in RA cells at 10^−7^ M (*p* = 0.043) (Figure 4B). Moreover, 10^−7^ M DAA significantly increased IL-10 concentration in OA and RA synoviocytes (OA: 10^−7^ M *p* = 0.02, 10^−9^ M compared to 10^−7^ M *p* = 0.29; RA: 10^−7^ M *p* = 0.004, 10^−9^ M compared to 10^−7^ M *p* = 0.005) (Figure 4B).

### 2.5. Effect of ENT1/2 Inhibiton

Since the transport of adenosine between intra- and extracellular space might influence its inflammatory effects, the impact of blocking adenosine transfer across the cell membrane was analyzed using the ENT1/ENT2 inhibitor DIP. This treatment did not modulate cytokine release in both OA and RA cells, and neither IL-6, TNF, nor IL-10 concentrations changed significantly compared to controls (Supplementary Materials Figure S1).

### 2.6. Effects of AR Agonists on IL-6 Release

**Figure 5:**
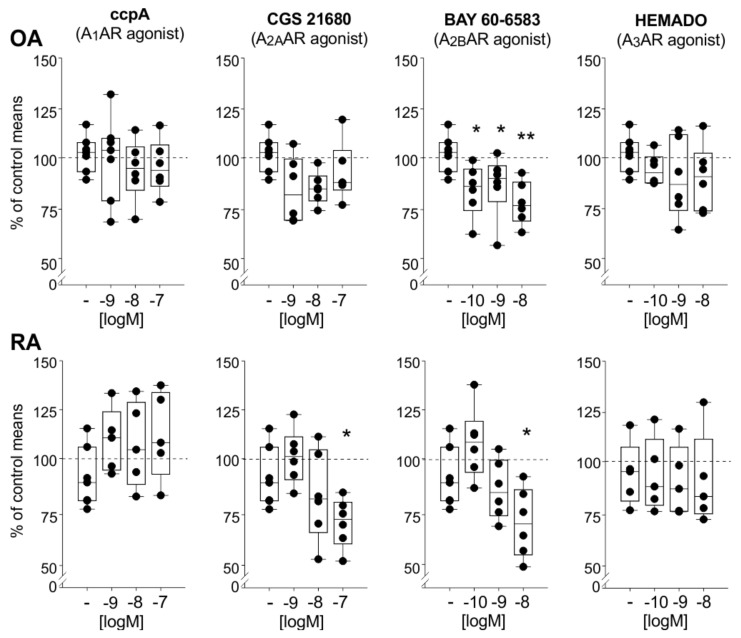
IL-6 release in OA and RA mixed synoviocyte cultures after activation of individual adenosine receptor subtypes by applying different AR agonists. Data are represented as box plots (as explained in the legend of Figure 1) (*n* = 5–6). Values are expressed as percent of untreated control (untreated control is represented as “-“; the mean of untreated controls is represented as dashed line = 100%). Significant *p*-values compared to untreated control are presented as * *p* ≤ 0.05 ** *p* ≤ 0.01 when compared to untreated controls. Abbreviations: see legend to Figure 1.

In order to study effects at specific ARs in mixed synoviocytes in vitro, we used agonists. IL-6 release in OA synoviocyte cultures was reduced only by the A_2B_AR agonist BAY 60-6583, but at all concentrations (10^−10^ M *p* = 0.014, 10^−9^ M *p* = 0.031, 10^−8^ M *p* = 0.002, Figure 5). In contrast, in RA synoviocytes, the treatment with the A_2A_AR agonist CGS 21680 also resulted in a significant IL-6 reduction; however, only at the higher concentration of 10^−8^ M (CGS 21680 *p* = 0.002, BAY 60-6583 *p* < 0.001) (Figure 5). The A_1_AR agonist ccpA and the A_3_AR agonist HEMADO showed no effect on either OA or RA synovial cell cultures regarding IL-6 release (Figure 5).

### 2.7. Effects of AR Agonists on TNF Release

**Figure 6:**
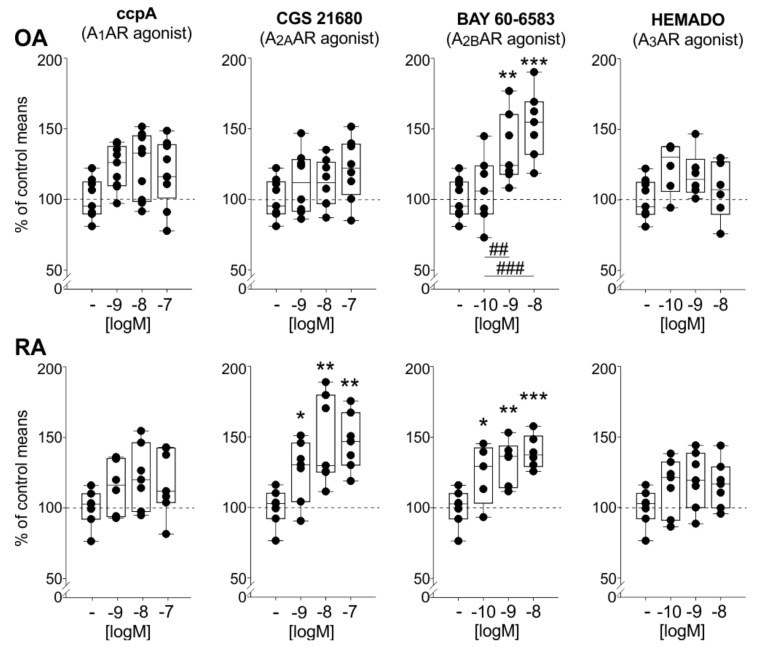
TNF release in OA and RA mixed synoviocyte cultures after activation of individual adenosine receptor subtypes by applying different AR agonists. Data are represented as box plots (as explained in the legend of Figure 1) (*n* = 5–6). Values are expressed as percent of untreated control (untreated control is represented as “-“; the mean of untreated controls is represented as dashed line = 100%). Significant *p*-values compared to untreated control are presented as p * ≤ 0.05. ** *p* ≤ 0.01. *** *p* ≤ 0.001 when compared to untreated controls or as ## *p* ≤ 0.05 ### *p* ≤ 0.001 when two treatment groups were compared. Abbreviations: see legend to Figure 1.

Similar to the effects on IL-6, the A_2A_AR agonist CGS 21680 significantly increased TNF release in RA but not in OA synoviocytes (10^−9^ M *p* = 0.039, 10^−8^ M *p* = 0.003, 10^−7^ M *p* = 0.002) (Figure 6). However, the A_2B_AR agonist BAY 60-6583 significantly enhanced the TNF levels in both OA ad RA synovial cell cultures (OA: 10^−9^ M *p* = 0.007, 10^−8^ M *p* < 0.001, 10^−10^ M compared to 10^−9^ M *p* = 0.023, 10^−10^ M compared to 10^−8^ M *p* < 0.001; RA: 10^−10^ M *p* = 0.021, 10^−9^ M *p* = 0.003, 10^−8^ M *p* < 0.001) (Figure 6). Furthermore, the A_1_AR agonist ccpA and the A_3_AR agonist HEMADO did not affect TNF release, neither in OA nor in RA synoviocyte cultures (Figure 6).

### 2.8. Effects of AR Agonists on IL-10 Release

**Figure 7:**
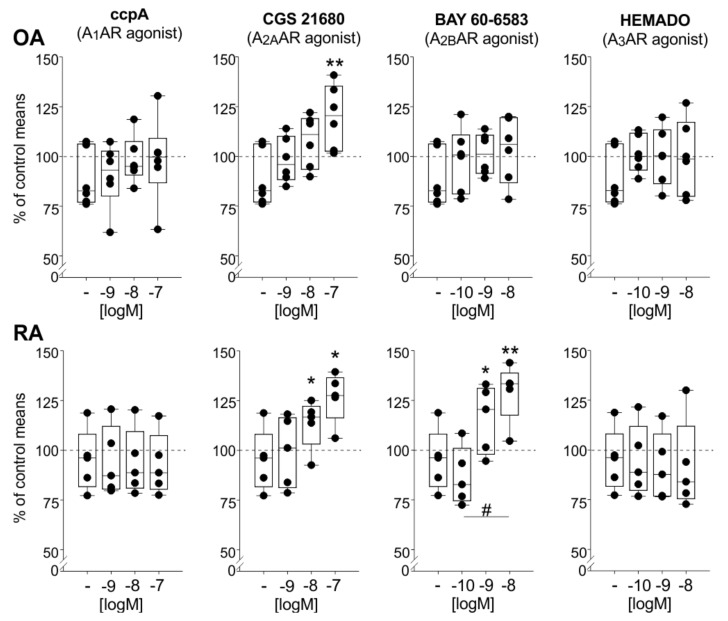
IL-10 release in OA and RA mixed synoviocyte cultures after activation of individual adenosine receptor subtypes by applying different AR agonists. Data are represented as box plots (as explained in the legend of Figure 1) (*n* = 5–6). Values are expressed as percent of untreated control (untreated control is represented as “-“; the mean of untreated controls is represented as dashed line = 100%). Significant *p*-values compared to untreated control are presented as * *p* ≤ 0.05 ** *p* ≤ 0.01 when compared to untreated controls or as # *p* ≤ 0.05 when two treatment groups were compared. Abbreviations: see legend to Figure 1.

In contrast to the effects on IL-6 and TNF, the treatment with CGS 21680 in high concentrations significantly elevated IL-10 release in both OA and RA synovial cells (OA: 10^−7^ M *p* = 0.005; RA: 10^−8^ M *p* = 0.022, 10^−7^ M *p* = 0.03) (Figure 7). In contrast, the A_2B_AR agonist BAY 60-6583 increased IL-10 synthesis only in RA but not in OA cells (10^−9^ M *p* = 0.036, 10^−8^ M *p* = 0.012, 10^−10^ M compared to 10^−8^ M *p* = 0.036) (Figure 7). Again, the A_1_AR agonist ccpA and the A_3_AR agonist HEMADO did not affect IL-10 release, neither in OA nor in RA synoviocyte cultures (Figure 7).

## 3. Discussion

A number of previous studies in humans and mice described that exogenously applied adenosine and inosine exert anti-inflammatory effects during RA pathogenesis [21], but it was never investigated whether synovial cells themselves are capable of producing these nucleosides, and if so, by which ARs autocrine effects might be exhibited. The present study demonstrates that both OA and RA synoviocytes express the complete enzymatic machinery to synthesize adenosine and inosine endogenously, which then mediate dual, predominantly anti-inflammatory but for the smaller part also pro-inflammatory, autocrine effects, mainly by activating the A_2A_- and A_2B_AR subtypes.

The initial step in this study was to analyze whether mixed synoviocytes from OA and RA patients express CD39 and CD73, the two cell surface enzymes that are known to convert ATP to adenosine, as well as ADA, being responsible for the conversion of adenosine to inosine [35]. We demonstrated that synovial cells from both patient groups expressed CD39 and CD73 to a comparable extent. This fits with the earlier studies mentioned above, demonstrating that regulatory T cells in RA synovial fluid express CD73 [30], and M2 macrophages in the blood of RA patients co-express CD39/CD73 [31]. Thus, the prerequisite for adenosine synthesis is given in mixed synovial cells. In order to convert adenosine to inosine, cells should express the enzyme ADA. We demonstrated that both OA and RA mixed synovial cells are ADA-positive. This is in line with previous studies that show that ADA was expressed by RA synovial fibroblasts [34], while similar data do not exist for OA fibroblasts or OA and RA synovial immune cells.

In addition, we demonstrated that the detected enzymes CD39, CD73 and ADA are active because synovial cells spontaneously release physiologically relevant amounts of adenosine and inosine. Nucleoside synthesis in synovial cells was never investigated before now. Until now, only endogenous catecholamine synthesis in mixed synovial cells from OA and RA patients has been described in our earlier studies [8,9]. However, OA synoviocytes produced significantly higher nucleoside concentrations compared to RA cells. The reason for this difference might be a decreased functionality of CD39 and CD73, or an elevated expression of adenosine- and inosine-degrading enzymes in RA due to the highly inflammatory situation [36]. In a chronic inflammatory microenvironment, which is accompanied by hypoxia, mitochondrial dysfunction and in turn decreased ATP synthesis occurs. This might lead to reduced adenosine levels as described by other studies [21]. Additionally, it has been reported that ADA activity is higher in RA synovial fluid compared to healthy or OA synovial fluids [37,38]. This could lead to the reduction in adenosine concentrations in RA tissue.

One prerequisite for a possible adenosine effect, besides the presence of adenosine itself, is the existence of ARs on target cells [21]. In general, all AR subtypes were detectable in both OA and RA synovium. This confirmed some results of our earlier study that demonstrated that isolated mixed synovial cells expressed all AR subtypes [9]. However, the expression levels of the different AR subtypes in synovial sections were different between OA and RA. In particular, the expression of A_2A_AR and A_2B_AR seemed to be more pronounced in RA synovial tissue. Until now, no study compared the expression of the different AR subtypes in the synovial tissue from OA and RA patients at the protein level. One possible reason for the elevated AR expression levels in RA might be the highly inflammatory microenvironment. Indeed, TNF, IL-β and LPS have been shown to upregulate A_2A_AR expression in human and equine monocytes [39,40]. This might be a compensatory mechanism for a higher ADA expression and function [37,38].

As the first evidence for the possible autocrine effects of endogenously synthesized adenosine, the release of the most prominent RA-related cytokines, namely IL-6, TNF and IL-10, were quantified after blocking CD73 activity, which is the rate-limiting step of adenosine production [35]. In general, we observed that RA synoviocytes release higher cytokine concentrations than OA cells, but the differences were not significant due to naturally occurring variation in human primary cultures [41,42]. A further reason for these differences might be the fact that the source of RA synoviocytes is a highly inflamed synovium containing more immune cells, while only a low-grade inflammation is present in OA synovium [43]. The fact that CD73 inhibition, causing an adenosine deficit, led to an increased pro-inflammatory (IL-6 and TNF) and decreased anti-inflammatory (IL-10) cytokine release, but ADA inhibition, leading to adenosine accumulation, resulted in the opposite, suggests that mainly endogenously released adenosine, and not inosine, is responsible for the anti-inflammatory effects in the synovium. This confirms the findings of earlier studies that show that adenosine treatment of RA synovial cells results in the inhibition of pro-inflammatory cytokines [22,23,24,25]. Moreover, it is astonishing that RA synoviocytes responded to CD73 or ADA inhibition in a similar manner as OA cells even though the concentration of endogenously produced adenosine or inosine was much lower in RA culture. This could be explained by a potentially higher nucleoside sensitivity of RA cells due to stronger A_2A_- and A_2B_AR expression or signal transduction.

Apparently, ENT1 and ENT2 do not influence the intra- and extracellular adenosine concentrations. The most likely explanation for this could be that ENT1/2 activity was balanced regarding adenosine transport between extra-and intracellular space without any significant gradient between these compartments. This result suggests that no ENT1/2 dysregulation takes place in OA or RA synovial cells during inflammation, although the opposite has been reported in studies investigating inflammatory bowel disease in mice, where ENT1/2 inhibition dampened intestinal inflammation [44]. In addition, this result also indicates that no intracellular adenosine, and accordingly its transport to extracellular space, is required to achieve anti-inflammatory effects.

The AR agonist treatments resulted in clear anti-inflammatory effects because the release of IL-6 was significantly reduced but IL-10 synthesis was induced. On the other hand, the same treatments also induced TNF, thus, ARs also mediate pro-inflammatory effects at the same time. We demonstrated that mainly the A_2A_- and A_2B_AR subtypes were responsible for these effects. The latter is in line with previous studies that showed that the majority of anti-inflammatory effects caused by exogenously applied adenosine was mediated by exactly these AR subtypes [22,23,24,25]. However, A_3_AR has also been described to exert anti-inflammatory effects in the RA synovium [45], which could not be confirmed in our study. One possible reason for this discrepancy might be that the concentration of endogenously synthesized adenosine in our study is lower and A_3_ARs possess lower affinity to adenosine compared to A_2A_Rs [17]. Therefore, mainly the A_2A_ARs have been targeted by the released adenosine.

The fact that the pro-inflammatory cytokine IL-6 was inhibited but the anti-inflammatory IL-10 was induced by A_2A_AR agonists is in agreement with the existing literature that shows similar effects in synoviocytes and peripheral lymphocytes of RA patients after adenosine or AR agonist application [9,22,23,24]. However, opposite to most previous studies, TNF release increased after the activation of A_2A_ARs. Two reasons might be responsible for this phenomenon. Firstly, the majority of former studies performed their experiments under hyperoxic conditions and not under hypoxia [23,46]. Secondly, AR desensitization due to continuous stimulation can lead to uncoupling of the receptors from the stimulatory Gαs protein, being the dominant signaling partners of the A_2A_ARs, and following coupling to other G proteins, such as the inhibitory Gαi [47]. Thus, TNF increase is likely the consequence of a pro-inflammatory AR switch, as described by us earlier for G protein coupled receptors in the synovium [9].

Interestingly, a similar TNF inhibition did not occur after ADA treatment. This might be due to the specificity of the AR agonists used (https://www.tocris.com/pharmacology/adenosine-receptors, accessed on 16 August 2021). Endogenously synthesized adenosine can in principle act via all AR subtypes expressed and the sum of the effects is anti-inflammatory because TNF was also reduced. In contrast, targeting exactly one AR subtype by applying a specific agonist does not represent the same situation. Therefore, we believe that rather, the natural ligand adenosine or a subtype specific synthetic agonist should be considered for therapy options.

The fact that most effects of inhibitors and agonists were stronger in RA than in OA synoviocytes can be explained by the differences in the inflammatory pattern. It is well known that RA is a primary inflammatory systemic disorder and in contrast to OA, much higher pro-inflammatory cytokine concentrations are present in the synovial tissue or fluid [14]. Thus, it is not surprising that that the effects of adenosine are not as pronounced as in RA. However, adenosine was also able to exert beneficial effects in OA synovial cells and should therefore be investigated in more detail in order to also develop a potent therapeutic strategy for OA.

One limitation of the present study might be that we did not further analyze the different cell types in mixed synoviocyte cultures specifically; however, this could also be seen as a strength of our investigations, because all cell types interact in the synovial tissue [48]. Therefore, we believe that analyzing the net effect of all cell types together provides a more holistic picture relevant to the clinical situation. Furthermore, in an in vitro synoviocyte model, it is not possible to investigate all factors playing a role in vivo at the same time. Besides adenosine, further factors play a role in synovial inflammation during RA and OA pathogenesis, such as the cytokines TNF or IL-1β, but also the major matrix-degrading enzymes MMP-1, -2, -3, -9, and -13 [49]. So far, only one study showed that anti-TNF therapy had no effect on the serum ADA level of RA patients compared to those who received DMARD therapy [50]. Another study reported a significant positive correlation between MMP-9 concentration and ADA activity in the synovial fluid of RA patients [51]. However, the causality between other factors and clinical phenotype has not yet been investigated in greater detail. Thus, the interaction between all these inflammatory microenvironment-related factors and endogenous adenosine/inosine has to be examined in future studies. Moreover, in most studies on synovial cell cultures, investigators take a maximum of eight to ten patients (10-20 years earlier, only three to five patients). Necessarily, all questions as to the influence of age of patients, medication, biological sex, menopausal status, or duration of the disease do not represent the conditions in normal synovium or in RA/OA patients with very early disease, because synovium will be most often taken during joint replacement surgery in a chronic phase. This represents a limitation to our studies.

## 4. Materials and Methods

### 4.1. Patients

Synovial tissue from patients with OA and RA was obtained during knee joint replacement surgery (patient characteristics are given in Table 1). Patients between 56 and 84 years were included in this study (age range of patients approved by the ethics committee: 18-85 years, inclusion criteria). The diagnosis of RA was based on the established criteria according to the American College of Rheumatology (formerly, the American Rheumatism Association) [52]. We excluded patients with joint infections. Patients were informed about the purpose of the study and gave their written consent. The Ethics Committee of the University of Regensburg approved the project (number 13-101-0135). All experiments were performed in accordance with relevant guidelines and regulations.

### 4.2. Synovial Tissue

Synovial tissue from RA and OA patients was obtained immediately after opening the knee joint capsule. Pieces of synovial tissue of up to 9 cm^2^ were excised. One part of the synovial tissue specimen was fixed for immunohistochemical analyses using 3.7% paraformaldehyde (PFA; Merck, Darmstadt, Germany), then infiltrated with increasing concentrations of sucrose (10–30%; Merck, Darmstadt, Germany), embedded in Tissue-Tek (Sakura Sakura Finetek, Zoeterwoude, The Netherlands), and cryosectioned at 6–8 μm.

### 4.3. Mixed Synovial Cells

For functional in vitro studies, the remaining tissue pieces were minced and placed in Dispase I (Roche Diagnostics, Penzberg, Germany). Digestion was carried out for at least 1 h at 37 °C on a shaking platform. The resulting suspension was filtered (70 µm) and spun at 300× *g* for 10 min. The pellet was then treated with erythrocyte lysis buffer (Merck, Darmstadt, Germany) for 5 min and recentrifuged for 10 min at 300× *g*. The pellet was resuspended in RPMI 1640 (Sigma-Aldrich, Taufkirchen, Germany) with 10% fetal calf serum (FCS; Thermo Fisher Scientific, Darmstadt, Germany). The obtained mixed synovial cells contain fibroblasts, macrophages, lymphocytes, and dendritic cells, as demonstrated in an earlier study [8]. For stimulation, 50.000 mixed synovial cells per well were transferred into 96-well culture plates and cultivated physioxic conditions (1% O_2_). After overnight incubation, cells were treated with different compounds, as described above. For immunocytochemical studies, untreated cells cytospinned and fixed with 3.7% PFA. Slides were then stored at −20 °C until analysis.

### 4.4. Immunostainings

In order to detect CD39 and CD73 co-expression by immunocytochemistry, fixed synovial cells were stained for these cell surface ectonucleotidases using the primary antibodies rabbit anti-CD39 (ab178572, Abcam, Cambridge, UK) and mouse anti-CD73 (ab133582, Abcam, Cambridge, UK). The expression of ADA, ENT1, ENT2, and ARs was investigated by immunohistochemical staining of synovial tissue samples using the primary antibodies rabbit anti-ADA (NBP1-90361, Novus Biologicals, Cambridge, UK), rabbit anti-ENT1 (NBP1-84838, Novus Biologicals, Cambridge, UK), rabbit anti-ENT2 (ab48595, Abcam, Cambridge, UK), rabbit anti-A_1_AR (ab124780, Abcam, Cambridge, UK), rabbit anti-A_2A_AR (ab260032, Abcam, Cambridge, UK), rabbit anti-A_2B_AR (LS-C20310, LSBio, via Biozol, Eching, Germany), and rabbit anti-A_3_AR (LS-A686, LSBio, via Biozol, Eching, Germany). After blocking (10% bovine serum albumine, 10% chicken serum, and 10% goat serum), cytospin or tissue slides were incubated with primary antibodies overnight at 4 °C. Primary staining was visualized using Alexa Fluor-labeled secondary antibodies (goat anti-rabbit Alexa Fluor 594 or goat anti-mouse or anti-rabbit Alexa Fluor 488; Thermo Fisher/Life technologies, Schwerte, Germany). Cell nuclei were counterstained with DAPI (Merck, Darmstadt, Germany). Slides without primary antibody served as negative controls (Supplementary Materials Figure S2).

### 4.5. Adenosine and Inosine Quantification

Spontaneous adenosine as well as inosine release was determined 24 h after cell seeding by HPLC as described earlier [53,54].

### 4.6. Synovial Cell Stimulation

After overnight incubation with standard medium, synoviocytes were treated with the CD73 inhibitor A,B-Methylenadenosine 5’diphosphate (AMPCP), the ADA inhibitor 1-Deazaadenosine (DAA) and the ENT1/2 inhibitor Dipyridamole (DIP). While AMPCP suppresses adenosine synthesis, DAA keeps the adenosine level high by preventing the conversion of adenosine to inosine. ENT1/2 are responsible for the transport of adenosine between the intra- and extracellular space.

To identify the AR subtypes responsible for possible adenosine-dependent effects, different selective AR subtype agonists (A_1_AR agonist 6-chloro-N6-cyclopentyladenosine (ccpA), A_2A_AR agonist CGS 21680 hydrochloride (CGS 21680), A_2B_AR agonist BAY 60–6583, A_3_AR agonist 2-(1-Hexynyl)-N-methyladenosine (HEMADO), all from Tocris Bioscience Wiesbaden-Nordenstadt, Germany, were used. After treatments for 24 h under physioxia, cell culture supernatants were collected and released. IL-6, TNF, and IL-10 concentrations were quantified by ELISA and Luminex assays.

### 4.7. Data Analysis

All experiments were carried out with synovial cells of at least five patients. Data are presented as box plots, as % of non-treated controls due to naturally occurring variation in primary culture. To test for normality, the Kolmogorov–Smirnov test was used. Differences between groups were calculated using the non-parametric ANOVA (when data were normally distributed) or ANOVA on ranks (when normality was not given) followed by the Bonferroni or Student–Newman–Keuls correction method (pairwise comparison of all groups), as suggested for the respective analysis by the statistics software (SigmaPlot V.11, Systat Software, Erkrath, Germany). *p* values less than 0.05 were considered significant.

## 5. Conclusions

Taken together, the present study clearly demonstrates that OA and RA synoviocytes express the full functional enzymatic machinery necessary for adenosine synthesis, namely CD39 and CD73.

Since the inhibition of CD73 resulted in clear pro-inflammatory effects, we speculate that CD73 might be a novel therapeutic target and promoting its activity might represent a novel treatment option. It has been shown for an in vitro microvascular endothelial model that the transcriptional activation of functional CD73 was induced by its product adenosine [55]. In the synovium, a similar positive feedback loop might result in the perpetuation of anti-inflammatory adenosine effects. We believe that the inhibition of ADA and the subsequent adenosine accumulation could lead to an increased CD73 activity.

Besides adenosine feedback regulation, hypoxia has been shown to impair CD73 function mediated by hypoxia-inducible factor-1 (HIF-1) in epithelial cells [21,56]. Since HIF-1 expression is increased in an inflamed synovium [57], we hypothesize that inhibiting HIF-1 would result in a functional rehabilitation of CD73 and in turn in elevated adenosine synthesis. Methotrexate, a standard and frequently used disease-modifying drug in RA therapy, has been described to increase adenosine levels besides numerous further possible mechanisms of action [58]. However, quite obviously, the adenosine induction by methotrexate is not sufficient to create or maintain the anti-inflammatory state. Therefore, further research is required to investigate whether CD73 induction can be induced in RA synovial cells using ADA and/or HIF-1 blocking drugs in addition to standard strategies.

In addition, we demonstrated that endogenously released adenosine exerted its anti-inflammatory effects mainly via A_2A_- and A_2B_ARs. Therefore, an alternative activation of these receptors using specific synthetic agonists also represents an attractive approach for the treatment of synovial inflammation in both OA and RA.

All things considered, we conclude that stimulating the CD39/CD73 enzymatic machinery and, at the same time, inhibiting ADA activity in the synovial tissue could be a useful addition to existing standard medications. This concept might not only represent a promising anti-inflammatory therapeutic strategy for RA patients but could also be beneficial for OA patients.

## Figures and Tables

**Table 1:** Characteristics of patients under study.

Patient Characteristics	Osteoarthritis	Rheumatoid Arthritis
Number	18	12
Mean age (range) (yr)	67.5 (56–84)	62.4 (56–79)
Number of women/men, n (%)	8/10 (44/56)	10/2 (83/17)
C-reactive protein (mg/l)	2.08 ± 1.88	11.44 ± 10.99
Medication		
Daily Prednisolone (mg)	n.a.	5.9
Prednisolone, n (%)	n.a.	9 (75)
Methotrexate, n (%)	n.a.	8 (67)
Leflunomide, n (%)	n.a.	1 (8)
Sulfasalazine, n (%)	n.a.	2 (17)
Hydroxychloroquinel, n (%)	n.a.	2 (17)
NSAID, n (%)	17 (94)	12 (100)
Steroids (other than Prednisolone), n (%)	n.a.	n.a
Opiod analgesics, n (%)	1 (6)	2 (17)
Biologicals, n (%)	n.a.	3 (25)

Abbreviations: yr—years, n—number, n.a.—not applicable, NSAID—non-steroidal anti-inflammatory drug.

## Data Availability

The data presented in this study are available on request from the corresponding author.

## Supplementary Materials

The following are available online at https://www.mdpi.com/article/10.3390/ijms22168956/s1.

## Abbreviations

ADA	Adenosine deaminase
Ado	Adenosine
AMP	Adenosine monophosphate
AMPCP	A: B-Methylenadenosine 5‘ diphosphate
AR	Adenosine receptor
ATP	Adenosine triphosphate
BAY	BAY 60-6583
ccPA	2-chloro-N6-cyclopentyladenosine
CD39	Ectonucleoside triphosphate diphosphohydrolase 1
CD73	Ecto 5′ nucleotidase
CGS	CGS 21680
DAA	1-Deazaadenosine
DAPI	4′,6-diamidino-2-phenylindole
DIP	Dipyridamole
DMARDs	Disease-modifying antirheumatic drugs
ENT1	Equilibrative Nucleoside Transporter 1
ENT2	Equilibrative Nucleoside Transporter 2
FCS	Fetal calf serum
HEMADO	2-(1-Hexynyl)-N-methyladenosine
HIF-1	Hypoxia-inducible factor-1
HPLC	High pressure liquid chromatography
IL-6	Interleukin 6
IL-10	Interleukin 10
Ino	Inosine
MMP	Matrix metalloproteinase
N	Number
n.a.	Not applicable
NSAID	Non-steroidal anti-inflammatory drug
OA	Osteoarthritis
PFA	Paraformaldehyde
RA	Rheumatoid arthritis
TNF	Tumor necrosis factor
yr	Years

## References

[1] Aletaha D., Smolen J.S. (2018). Diagnosis and Management of Rheumatoid Arthritis: A Review. JAMA.

[2] Sweeney S.E., Firestein G.S. (2004). Rheumatoid arthritis: Regulation of synovial inflammation. Int. J. Biochem. Cell Biol..

[3] Scott D.L., Wolfe F., Huizinga T.W. (2010). Rheumatoid arthritis. Lancet.

[4] Yu M.B., Firek A., Langridge W.H.R. (2018). Predicting methotrexate resistance in rheumatoid arthritis patients. Inflammopharmacology.

[5] Deane K.D., Demoruelle M.K., Kelmenson L.B., Kuhn K.A., Norris J.M., Holers V.M. (2017). Genetic and environmental risk factors for rheumatoid arthritis. Best Pract. Res. Clin. Rheumatol..

[6] Schönfeld C., Pap T., Neumann E., Müller-Ladner U. (2015). Fibroblasts as pathogenic cells in rheumatic inflammation. Z. Fur Rheumatol..

[7] McInnes I.B., Schett G. (2017). Pathogenetic insights from the treatment of rheumatoid arthritis. Lancet.

[8] Capellino S., Cosentino M., Wolff C., Schmidt M., Grifka J., Straub R.H. (2010). Catecholamine-producing cells in the synovial tissue during arthritis: Modulation of sympathetic neurotransmitters as new therapeutic target. Ann. Rheum. Dis..

[9] Jenei-Lanzl Z., Zwingenberg J., Lowin T., Anders S., Straub R.H. (2015). Proinflammatory receptor switch from Gαs to Gαi signaling by β-arrestin-mediated PDE4 recruitment in mixed RA synovial cells. Brain Behav. Immun..

[10] McInnes I.B., Buckley C.D., Isaacs J.D. (2016). Cytokines in rheumatoid arthritis—Shaping the immunological landscape. Nat. Rev. Rheumatol..

[11] McInnes I.B., Schett G. (2007). Cytokines in the pathogenesis of rheumatoid arthritis. Nat. Rev. Immunol..

[12] Schett G., Emery P., Tanaka Y., Burmester G., Pisetsky D.S., Naredo E., Fautrel B., van Vollenhoven R. (2016). Tapering biologic and conventional DMARD therapy in rheumatoid arthritis: Current evidence and future directions. Ann. Rheum. Dis..

[13] Vlachogiannis N.I., Gatsiou A., Silvestris D.A., Stamatelopoulos K., Tektonidou M.G., Gallo A., Sfikakis P.P., Stellos K. (2020). Increased adenosine-to-inosine RNA editing in rheumatoid arthritis. J. Autoimmun..

[14] Scanzello C.R. (2017). Role of low-grade inflammation in osteoarthritis. Curr. Opin. Rheumatol..

[15] Milne G.R., Palmer T.M. (2011). Anti-inflammatory and immunosuppressive effects of the A2A adenosine receptor. Sci. World J..

[16] Odashima M., Bamias G., Rivera-Nieves J., Linden J., Nast C.C., Moskaluk C.A., Marini M., Sugawara K., Kozaiwa K., Otaka M. (2005). Activation of A2A adenosine receptor attenuates intestinal inflammation in animal models of inflammatory bowel disease. Gastroenterology.

[17] Fredholm B.B., AP I.J., Jacobson K.A., Linden J., Müller C.E. (2011). International Union of Basic and Clinical Pharmacology. LXXXI. Nomenclature and classification of adenosine receptors—An update. Pharmacol. Rev..

[18] Sattin A., Rall T.W. (1970). The effect of adenosine and adenine nucleotides on the cyclic adenosine 3’, 5’-phosphate content of guinea pig cerebral cortex slices. Mol. Pharmacol..

[19] Vecchio E.A., White P.J., May L.T. (2017). Targeting Adenosine Receptors for the Treatment of Cardiac Fibrosis. Front. Pharmacol..

[20] Stamp L.K., Hazlett J., Roberts R.L., Frampton C., Highton J., Hessian P.A. (2012). Adenosine receptor expression in rheumatoid synovium: A basis for methotrexate action. Arthritis Res. Ther..

[21] Cronstein B.N., Sitkovsky M. (2017). Adenosine and adenosine receptors in the pathogenesis and treatment of rheumatic diseases. Nat. Rev. Rheumatol..

[22] Campo G.M., Avenoso A., D’Ascola A., Nastasi G., Micali A., Puzzolo D., Pisani A., Prestipino V., Scuruchi M., Calatroni A. (2013). Combined treatment with hyaluronan inhibitor Pep-1 and a selective adenosine A2 receptor agonist reduces inflammation in experimental arthritis. Innate Immun..

[23] Flögel U., Burghoff S., van Lent P.L., Temme S., Galbarz L., Ding Z., El-Tayeb A., Huels S., Bönner F., Borg N. (2012). Selective activation of adenosine A2A receptors on immune cells by a CD73-dependent prodrug suppresses joint inflammation in experimental rheumatoid arthritis. Sci. Transl. Med..

[24] Li Q.H., Xie W.X., Li X.P., Huang K.T., Du Z.H., Cong W.J., Zhou L.H., Ye T.S., Chen J.F. (2015). Adenosine A2A Receptors Mediate Anti-Inflammatory Effects of Electroacupuncture on Synovitis in Mice with Collagen-Induced Arthritis. Evid. Complementary Altern. Med. eCAM.

[25] Magni G., Ceruti S. (2020). Adenosine Signaling in Autoimmune Disorders. Pharmaceuticals.

[26] Muz B., Khan M.N., Kiriakidis S., Paleolog E.M. (2009). Hypoxia. The role of hypoxia and HIF-dependent signalling events in rheumatoid arthritis. Arthritis Res. Ther..

[27] Fishman P., Cohen S. (2016). The A3 adenosine receptor (A3AR): Therapeutic target and predictive biological marker in rheumatoid arthritis. Clin. Rheumatol..

[28] Novotný J. (2015). Adenosine and its role in physiology. Ceskoslovenska Fysiol..

[29] Giatromanolaki A., Kouroupi M., Pouliliou S., Mitrakas A., Hasan F., Pappa A., Koukourakis M.I. (2020). Ectonucleotidase CD73 and CD39 expression in non-small cell lung cancer relates to hypoxia and immunosuppressive pathways. Life Sci..

[30] Zhang R., Miao J., Zhang K., Zheng Z., Zhu P. (2019). Increased percentage and defective inhibitory function of CD4(+)CD25(−)FOXP3(+) T cells in synovial fluid of patients with rheumatoid arthritis. Xi Bao Yu Fen Zi Mian Yi Xue Za Zhi Chin. J. Cell. Mol. Immunol..

[31] Ohradanova-Repic A., Machacek C., Charvet C., Lager F., Le Roux D., Platzer R., Leksa V., Mitulovic G., Burkard T.R., Zlabinger G.J. (2018). Extracellular Purine Metabolism Is the Switchboard of Immunosuppressive Macrophages and a Novel Target to Treat Diseases With Macrophage Imbalances. Front. Immunol..

[32] Baldwin S.A., Beal P.R., Yao S.Y., King A.E., Cass C.E., Young J.D. (2004). The equilibrative nucleoside transporter family, SLC29. Pflug. Arch. Eur. J. Physiol..

[33] Van der Weyden M.B., Kelley W.N. (1976). Human adenosine deaminase. Distribution and properties. J. Biol. Chem..

[34] Nakamachi Y., Koshiba M., Nakazawa T., Hatachi S., Saura R., Kurosaka M., Kusaka H., Kumagai S. (2003). Specific increase in enzymatic activity of adenosine deaminase 1 in rheumatoid synovial fibroblasts. Arthritis Rheum..

[35] Szabo C., Pacher P. (2012). The outsiders: Emerging roles of ectonucleotidases in inflammation. Sci. Transl. Med..

[36] Coates L.C., FitzGerald O., Helliwell P.S., Paul C. (2016). Psoriasis, psoriatic arthritis, and rheumatoid arthritis: Is all inflammation the same?. Semin. Arthritis Rheum..

[37] Yuksel H., Akoğlu T.F. (1988). Serum and synovial fluid adenosine deaminase activity in patients with rheumatoid arthritis, osteoarthritis, and reactive arthritis. Ann. Rheum. Dis..

[38] Zakeri Z., Izadi S., Niazi A., Bari Z., Zendeboodi S., Shakiba M., Mashhadi M., Narouie B., Ghasemi-Rad M. (2012). Comparison of adenosine deaminase levels in serum and synovial fluid between patients with rheumatoid arthritis and osteoarthritis. Int. J. Clin. Exp. Med..

[39] Khoa N.D., Montesinos M.C., Reiss A.B., Delano D., Awadallah N., Cronstein B.N. (2001). Inflammatory cytokines regulate function and expression of adenosine A(2A) receptors in human monocytic THP-1 cells. J. Immunol..

[40] Sun W.C., Berghaus L.J., Moore J.N., Hurley D.J., Vandenplas M.L., Thompson R., Linden J. (2010). Lipopolysaccharide and TNF-alpha modify adenosine A(2A) receptor expression and function in equine monocytes. Vet. Immunol. Immunopathol..

[41] Altschuler S.J., Wu L.F. (2010). Cellular heterogeneity: Do differences make a difference?. Cell.

[42] Thomas S., Rouilly V., Patin E., Alanio C., Dubois A., Delval C., Marquier L.G., Fauchoux N., Sayegrih S., Vray M. (2015). The Milieu Intérieur study—An integrative approach for study of human immunological variance. Clin. Immunol..

[43] Robinson W.H., Lepus C.M., Wang Q., Raghu H., Mao R., Lindstrom T.M., Sokolove J. (2016). Low-grade inflammation as a key mediator of the pathogenesis of osteoarthritis. Nat. Rev. Rheumatol..

[44] Aherne C.M., Collins C.B., Rapp C.R., Olli K.E., Perrenoud L., Jedlicka P., Bowser J.L., Mills T.W., Karmouty-Quintana H., Blackburn M.R. (2018). Coordination of ENT2-dependent adenosine transport and signaling dampens mucosal inflammation. JCI Insight.

[45] Bar-Yehuda S., Silverman M.H., Kerns W.D., Ochaion A., Cohen S., Fishman P. (2007). The anti-inflammatory effect of A3 adenosine receptor agonists: A novel targeted therapy for rheumatoid arthritis. Expert Opin. Investig. Drugs.

[46] Ochaion A., Bar-Yehuda S., Cohen S., Amital H., Jacobson K.A., Joshi B.V., Gao Z.G., Barer F., Patoka R., Del Valle L. (2008). The A3 adenosine receptor agonist CF502 inhibits the PI3K, PKB/Akt and NF-kappaB signaling pathway in synoviocytes from rheumatoid arthritis patients and in adjuvant-induced arthritis rats. Biochem. Pharmacol..

[47] Daaka Y., Luttrell L.M., Lefkowitz R.J. (1997). Switching of the coupling of the beta2-adrenergic receptor to different G proteins by protein kinase A. Nature.

[48] Aarvak T., Natvig J.B. (2001). Cell-cell interactions in synovitis: Antigen presenting cells and T cell interaction in rheumatoid arthritis. Arthritis Res..

[49] Burrage P.S., Mix K.S., Brinckerhoff C.E. (2006). Matrix metalloproteinases: Role in arthritis. Front. Biosci. A J. Virtual Libr..

[50] Erer B., Yilmaz G., Yilmaz F.M., Koklu S. (2009). Assessment of adenosine deaminase levels in rheumatoid arthritis patients receiving anti-TNF-alpha therapy. Rheumatol. Int..

[51] Iwaki-Egawa S., Watanabe Y., Matsuno H. (2001). Correlations between matrix metalloproteinase-9 and adenosine deaminase isozymes in synovial fluid from patients with rheumatoid arthritis. J. Rheumatol..

[52] Arnett F.C., Edworthy S.M., Bloch D.A., McShane D.J., Fries J.F., Cooper N.S., Healey L.A., Kaplan S.R., Liang M.H., Luthra H.S. (1988). The American Rheumatism Association 1987 revised criteria for the classification of rheumatoid arthritis. Arthritis Rheum..

[53] Huang L.F., Guo F.Q., Liang Y.Z., Li B.Y., Cheng B.M. (2004). Simple and rapid determination of adenosine in human synovial fluid with high performance liquid chromatography-mass spectrometry. J. Pharm. Biomed. Anal..

[54] Straub R.H., Pongratz G., Günzler C., Michna A., Baier S., Kees F., Falk W., Schölmerich J. (2002). Immunoregulation of IL-6 secretion by endogenous and exogenous adenosine and by exogenous purinergic agonists in splenic tissue slices. J. Neuroimmunol..

[55] Narravula S., Lennon P.F., Mueller B.U., Colgan S.P. (2000). Regulation of endothelial CD73 by adenosine: Paracrine pathway for enhanced endothelial barrier function. J. Immunol..

[56] Synnestvedt K., Furuta G.T., Comerford K.M., Louis N., Karhausen J., Eltzschig H.K., Hansen K.R., Thompson L.F., Colgan S.P. (2002). Ecto-5’-nucleotidase (CD73) regulation by hypoxia-inducible factor-1 mediates permeability changes in intestinal epithelia. J. Clin. Investig..

[57] Hu F., Liu H., Xu L., Li Y., Liu X., Shi L., Su Y., Qiu X., Zhang X., Yang Y. (2016). Hypoxia-inducible factor-1α perpetuates synovial fibroblast interactions with T cells and B cells in rheumatoid arthritis. Eur. J. Immunol..

[58] Friedman B., Cronstein B. (2019). Methotrexate mechanism in treatment of rheumatoid arthritis. Jt. Bone Spine.

