# Supplementary material for: Anti-Inflammatory Effects of Endogenously Released Adenosine in Synovial Cells of Osteoarthritis and Rheumatoid Arthritis Patients
Source: Int J Mol Sci. 2021 Aug 19;22(16):8956. doi: 10.3390/ijms22168956 (PMC8396606; doi:10.3390/ijms22168956)
Supplement: Supplementary file 1 [file ijms-22-08956-s001.zip › ijms-1336470-supplementary.pdf]

International Journal of
Molecular Sciences

MDPI

Supplementary material to the Article

Anti-Inflammatory Effects of Endogenously Released Adenosine in Synovial Cells of Osteoarthritis and Rheumatoid Arthritis Patients

Rebecca Sohn [1§], Marius Junker [1§], Andrea Meurer [1], Frank Zaucke [1], Rainer H. Straub [2#], Zsuzsa Jenei-Lanzl [1*#]

[1] Dr. Rolf M. Schwiete Research Unit for Osteoarthritis, Department of Orthopedics (Friedrichsheim), University Hospital Frankfurt, Goethe University, 60528 Frankfurt / Main, Germany; rebecca.sohn@kgu.de (R.S.), marius.junker@kgu.de (M.J.), andrea.meurer@kgu.de (A.M.), frank.zaucke@kgu.de (F.Z.), zsuzsa.jenei-lanzl@kgu.de (Z.J-L.)

[2] Laboratory of Experimental Rheumatology and Neuroendocrine Immunology, Department of Internal Medicine, University Hospital Regensburg, 93053 Regensburg, Germanyrainer.straub@ukr.de (R.H.S)

[§] Contributed equally as first author

[#] Contributed equally as senior author

[*] Correspondence: zsuzsa.jenei-lanzl@kgu.de (Z.J-L.), Tel.: +49-69-6301-94-408

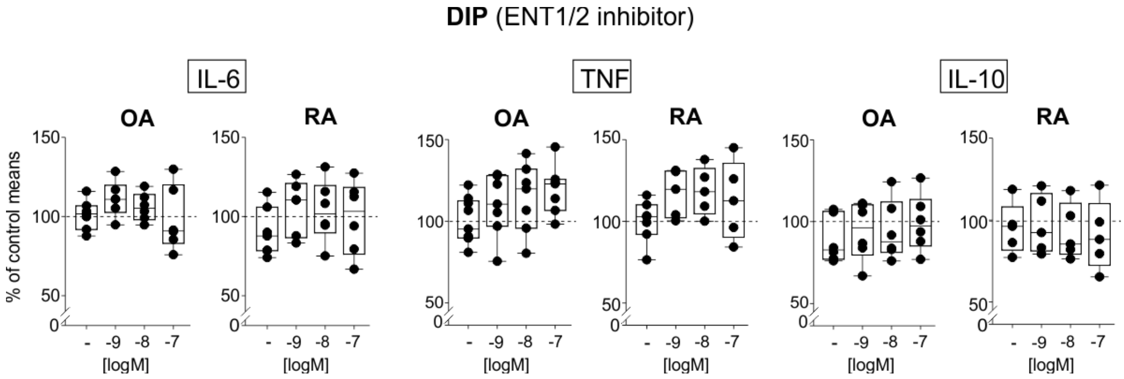

Supplementary Figure S1. Effect of ENT1/2 inhibiton. ENT1/2 inhibition in OA and RA mixed synoviocyte cultures using DIP (10^{-7} M to 10^{-9} M). Data are represented as box plots (as explained in the legend of Figure 1)(n=5-6). Values are expressed as percent of untreated control (untreated control is represented as “-“; the mean of untreated control is represented as dashed line = 100%).

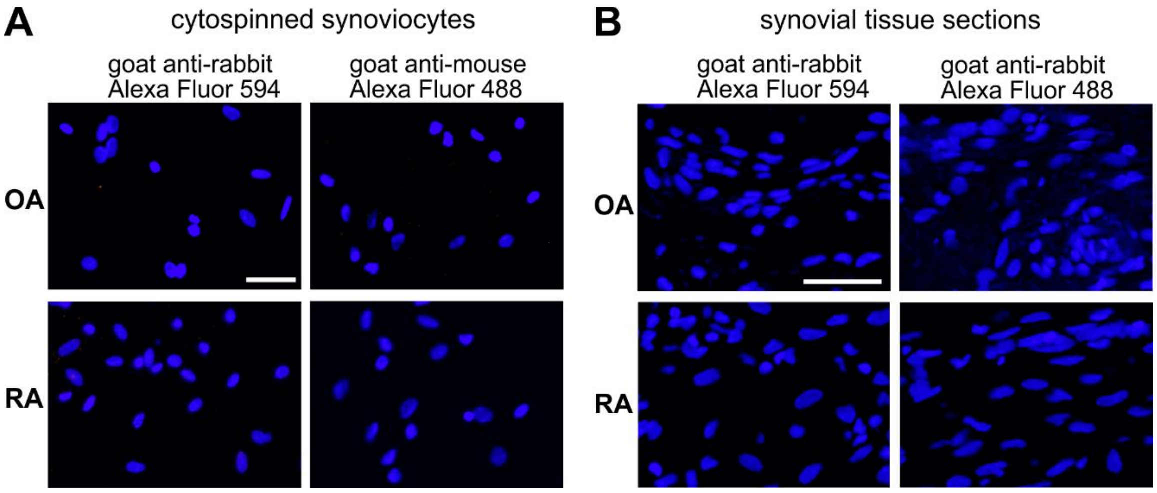

Supplementary Figure S2. Negative controls (without primary but with secondary antibodies) for the immunostainings of (A) cytospinned synoviocytes (bar: 25 µm) and (B) synovial tissue sections (bar: 50 µm) represented in Fig.1, Fig.2, and Fig.3 (goat anti-rabbit Alexa Fluor 594 for CD39, ENT1, ENT2, all ARs; goat anti-mouse Alexa Fluor 488 for CD73; goat anti-rabbit Alexa Fluor 488 for ADA). Cell nuclei were counterstained with DAPI (blue).

Abbreviations: OA - osteoarathritis,; RA – rheumatoid arthritis.
